# Supplementary material for: Splice-Junction-Based Mapping of Alternative Isoforms in the Human Proteome
Source: Cell Rep. Author manuscript; Available in PMC 2020 Jan 15. (PMC6961840; doi:10.1016/j.celrep.2019.11.026)
Supplement: 2 [file NIHMS1546469-supplement-2.zip › DF1/PXD009737/TestisTrypsin/TestisTrypsin_24_ART3_LEDHAPGPVPVPGPK.pdf]

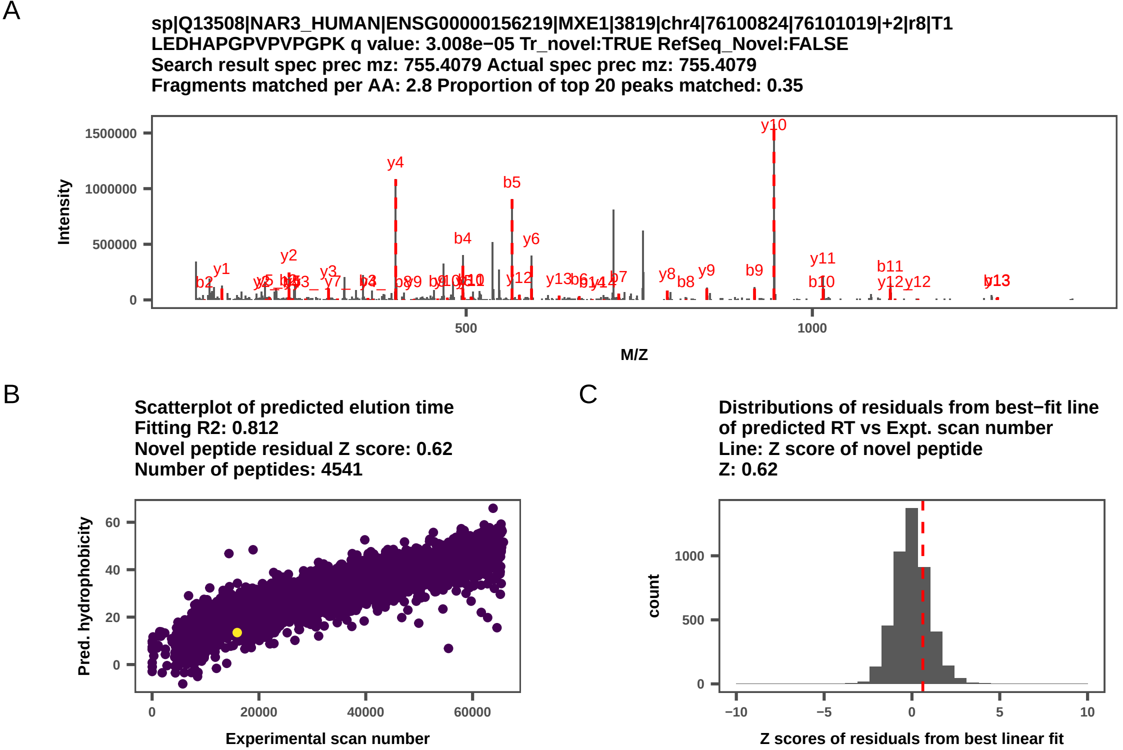
A
sp|Q13508|NAR3_HUMAN|ENSG00000156219|MXE1|3819|chr4|76100824|76101019|+2|r8|T1
LEDHAPGPVPVPGPK q value: 3.008e−05 Tr_novel:TRUE RefSeq_Novel:FALSE
Search result spec prec mz: 755.4079 Actual spec prec mz: 755.4079
Fragments matched per AA: 2.8 Proportion of top 20 peaks matched: 0.35
Intensity
1500000
1000000
500000
0
M/Z
500
1000
y10
y4
b5
b4
y6
y2
y1
y3
y7
b2
y12
y13
b7
y8
b8
y9
b9
y11
b10
b11
y12
B
Scatterplot of predicted elution time
Fitting R2: 0.812
Novel peptide residual Z score: 0.62
Number of peptides: 4541
Pred. hydrophobicity
60
40
20
0
0
20000
40000
60000
Experimental scan number
C
Distributions of residuals from best−fit line
of predicted RT vs Expt. scan number
Line: Z score of novel peptide
Z: 0.62
count
1000
500
0
−10
−5
0
5
10
Z scores of residuals from best linear fit
